# p16INK4a in cellular senescence

**DOI:** 10.18632/aging.100592

**Published:** 2013-08-17

**Authors:** Elisabeth Simboeck, Luciano Di Croce

**Affiliations:** ^1^ Centre for Genomic Regulation (CRG) and UPF, Dr. Aiguader 88, 08003 Barcelona, Spain; ^2^ INSERM, Institute of Human Genetics (IGH), CNRS UPR 1142, Montpellier, France; ^3^ Institució Catalana de Recerca i Estudis Avançats (ICREA), Pg. Lluis Companys 23, 08010 Barcelona, Spain

A crucial mechanism in the establishment of cellular senescence is the activation of the INK4/ARF locus, which is epigenetically regulated and under tight control of the Polycomb group (PcG) Trithorax group (TrxG) proteins [1]. In proliferating cells, the locus is silenced by Polycomb repressive complexes (PRCs), and the chromatin is enriched in H3K27me3 [2]. Upon senescence triggers, PRCs are displaced and the repressive H3K27me3 mark is removed. Instead, the Trithorax MLL1 complex is recruited and leads to deposition of H3K4me3 and transcriptional activation of the locus [1]. Correspondingly, it was shown that overexpression of PcG proteins delayed the onset of replicative senescence, while deletion of MLL1 bypassed OIS by repressing the INK4/ARF locus [1,3]. Interestingly, we recently discovered that transcriptional regulation of p16INK4a can occur independently of H3K4me3 and H3K27me3 and is instead dependent on the proper function of transcription factors [4]. While characterizing DPY30, a common member of all H3K4 histone methyltransferase (HMTase) complexes (including MLL1), we found that DPY30 is a crucial regulator of cell proliferation. Depletion of DPY30 in human fibroblasts, as well as in transformed cells, led to severe impairment of cell cycle progression and a senescence-like phenotype [4]. This included a flattened and enlarged morphology, elevated levels of reactive oxygen species (ROS), activation of DNA damage response pathways (DDR), increased SA-β-galactosidase activity, formation of senescence-associated heterochromatin foci (SAHFs) and increased p16INK4a expression levels.

**Figure 1:**
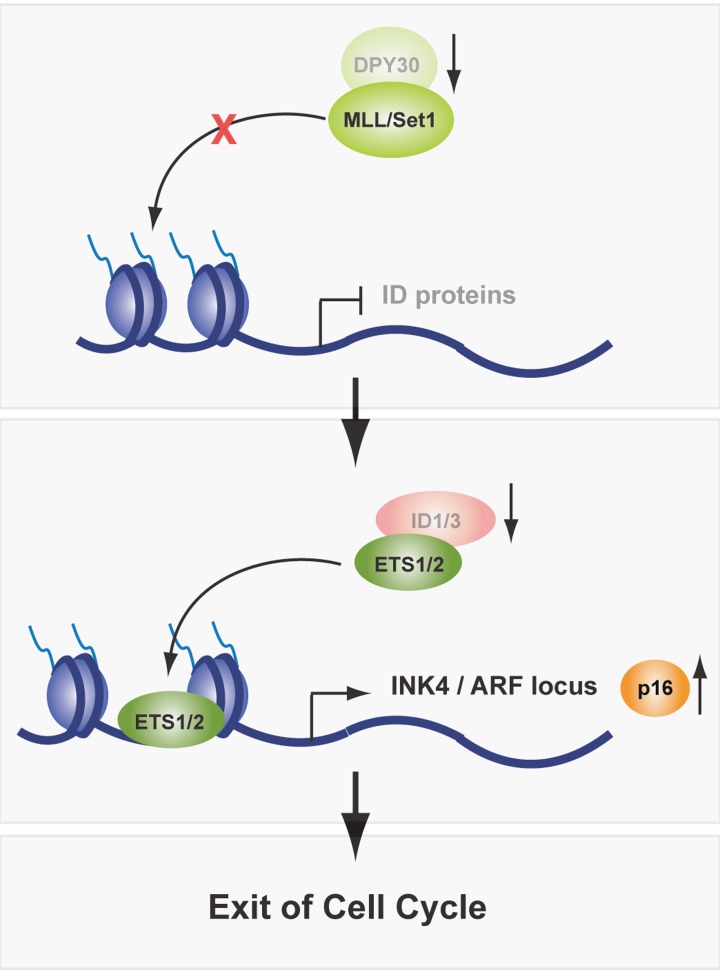
In aging, expression levels of DPY30 are decreased, which impairs the activity of the MLL/Set1 complex and downregulates ID protein expression. As a consequence, Ets1/2 transcription factors are free to bind to, and activate expression of the INK4/ARF locus. Thereafter, cells exit cell cycle and enter cellular senescence.

Although activation of INK4/ARF was in accordance with the observed senescence-like phenotype, it immediately raised the question how this activation takes place in the absence of an intact DPY30-MLL/Set1 complex. We wondered whether a H3K4-specific HTMase complex might still be active in the absence of DPY30. However, H3K4me3 levels were significantly reduced on the p16INK4a promoter. These results indicate that DPY30 is essential for an active HMTase complex, but that its action is not strictly required to activate p16INK4a expression. Nevertheless, we observed an active chromatin conformation at the p16INK4a promoter in DPY30 knockdown cells that was characterized by hyperacetylation of histones H3 and H4, as well as increased Ets1/2 occupancy, a transcription factor required for activation of the INK4/ARF locus [5]. To better understand how the loss of DPY30 can lead to a senescence-like phenotype, we screened for direct DPY30 target genes using a genome-wide approach that combined ChIP sequencing for DPY30 and H3K4me3 with expression arrays in wild-type and DPY30 knockdown cells. Interestingly, DPY30 target genes are predominantly involved in cell cycle and proliferation regulation. For one of the direct DPY30 targets, *i.e.* the ID proteins, we showed that their transcriptional silencing upon DPY30 depletion was partially responsible for the senescence-like phenotype, as reintroduction of ID1 and ID3 led to a partial rescue of the observed phenotype. Accordingly, p16INK4a was de-repressed, however independently of Polycomb activity and H3K27me3. ID proteins can negatively regulate the function of transcription factors, including Rb and Ets1/2. During cell cycle progression, Ets1/2 is phosphorylated and activated by MAP kinases. ID proteins bind to phosphorylated Ets1/2 and counter balance their activity [6]. In OIS, Ets1/2 are constitutively phosphorylated by oncogenic Ras/MAPK signaling and therefore overwrite the steady state controlled by ID proteins, resulting in p16INK4a expression [7]. In replicative senescence, p16INK4a is activated due to increased expression of Ets1/2 and decreased expression of ID proteins. However, how changes in Ets1/2 and ID protein expression during aging are regulated is still elusive. Preliminary observations in aged mouse keratinocytes and replicative senescent human fibroblasts suggest that decreased ID protein expression could be a direct consequence of lower DPY30 expression levels. Interestingly, the expression of other H3K4 HMTase complex members remained unchanged in aged cells. These findings indicate that INK4/ARF is regulated by the Ets1/2 transcription factor via the fine-tuning of ID protein expression by DPY30, which might be physiologically relevant in aging (Figure 1).

We speculate that the regulation of ID protein expression by DPY30 could also be implicated in tumorigenesis. In addition to a frequent deregulation of PcG and TrxG proteins in cancer, ID protein levels are elevated in some types of tumors, which was associated with disease severity and poor prognosis [8]. We wondered if elevated ID protein levels in cancer could result from deregulated DPY30-MLL/Set1 complex action. After DPY30 depletion in several cancer cell lines, we observed not only a decrease in ID protein expression but also that proliferation was compromised, suggesting that indeed ID protein expression is under control of DPY30 and that their overexpression could be beneficial for the proliferation rate in these transformed cells. However, whether upregulation of ID proteins by DPY30 in cancer follows the same axis as in aging, namely, the regulation of Ets1/2 activity and consequently of INK4/ARF expression, has not been determined and requires further studies.

Given the importance of understanding the molecular mechanisms involved in cellular senescence and tumorigenesis, we believe that these recent findings [4] unravel an additional part of this complex mosaic of pathways and suggest an alternative possibility of p16INK4a regulation. Further investigations on the transcriptional regulation and function of the ID proteins will help to further dissect their role in aging and could possibly unravel ID protein overexpression as a tumor marker and, importantly, as a potential target for designing novel anti-cancer treatments.
